# Evading plant immunity: feedback control of the T3SS in *Pseudomonas syringae*

**DOI:** 10.15698/mic2017.04.570

**Published:** 2017-03-17

**Authors:** Christopher Waite, Jörg Schumacher, Milija Jovanovic, Mark Bennett, Martin Buck

**Affiliations:** 1 Department of Life Sciences, Imperial College London, UK.

**Keywords:** gene regulation, feedback control, T3SS, HrpL, plants, pathogen, syringae

## Abstract

Microbes are responsible for over 10% of the global yield losses in staple crops such as wheat, rice, and maize. Understanding the decision-making strategies that enable bacterial plant pathogens to evade the host immune system and cause disease is essential for managing their ever growing threat to food security. Many utilise the needle-like type III secretion system (T3SS) to suppress plant immunity, by injecting effector proteins that inhibit eukaryotic signalling pathways into the host cell cytoplasm. Plants can in turn evolve resistance to specific pathogens via recognition and blocking of the T3SS effectors, so leading to an ongoing co-evolutionary ‘arms race’ between pathogen and host pairs. The extracytoplasmic function sigma factor HrpL co-ordinates the expression of the T3SS regulon in the leaf-dwelling *Pseudomonas syringae* and similar pathogens. Recently, we showed that association of HrpL with a target promoter directly adjacent to the *hrpL* gene imposes negative autogenous control on its own expression level due to overlapping regulatory elements. Our results suggest that by down-regulating T3SS function, this fine-tuning mechanism enables *P. syringae* to minimise effector-mediated elicitation of plant immunity.

The *hrp* and *hrc* genes required to build and regulate the T3SS of bacterial plant pathogens, plus many effector loci, are commonly found clustered together in a pathogenicity island. In a major subset of plant pathogens, the extra-cytoplasmic function (ECF) sigma factor HrpL co-regulates these 50 or so genes via a conserved promoter motif known as the *hrp*-box. The expression of HrpL itself is dependent on the alternative sigma-54 (σ^54^), necessitating a complex transcription initiation process: a hexameric bacterial enhancer binding protein (EBP) binds the promoter at a distal upstream activation sequence (UAS) before being brought into contact with the inactive RNA polymerase (RNAP)-σ^54^ holoenzyme via DNA looping. In *P. syringae*, transcription of *hrpL* is activated by a hetero-hexamer comprising the co-dependent EBPs, HrpR and HrpS, which are thought to respond to currently unknown environmental signals. Located within the *hrp*/*hrc* gene cluster, the *hrpL* gene shares a short, divergent promoter region (approximately 200 nt) with the HrpL-activated *hrpJ* operon (**Figure 1**). Given that the *hrpL* UAS is located approximately 100-150 nt upstream of the transcriptional start site, our objective was to determine whether the juxtaposition of the HrpRS and RNAP-HrpL binding sites leads to regulatory coupling between the expression levels of *hrpL* and the *hrpJ* operon, specifically in the model *P. syringae* pathovar* tomato* DC3000.

**Figure 1:**
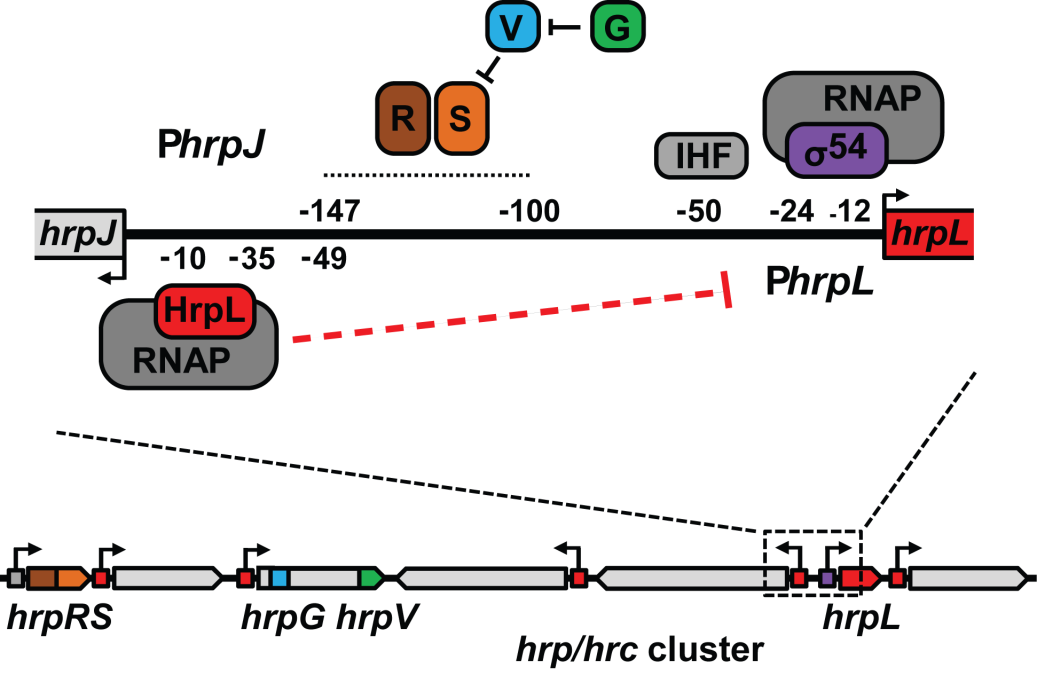
FIGURE 1: The regulation of *hrpL* transcription in *P. syringae*. Organisation of the bi-directional promoter region between *hrpL* and *hrpJ*, including known binding sites for σ^54^, IHF, and HrpRS. HrpS activator function is regulated antagonistically by HrpV and HrpG. HrpL promotes transcription of *hrpJ* and other *hrp*/*hrc* operons via the *hrp*-box (red boxes). This study proposes a mechanism of negative autogenous control dependent on RNAP-HrpL binding at the *hrpJ* promoter (dashed red line). Figure reproduced from Waite *et al.* 2017 (doi: 10.1128/mBio.02273-16) under the Creative Commons CC BY 4.0 license.

Data from a plasmid-borne GFP reporter fusion and RNA sequencing suggested that the *hrpL* promoter (P*hrpL*) is subject to a strong negative feedback, since its activity increased in a Δ*hrpL* deletion mutant. This observation was not unexpected given that a small regulatory protein belonging to the HrpL regulon, HrpV, is known to repress HrpS during activation of P*hrpL*. However, the increase in P*hrpL* activity was far more exaggerated in the Δ*hrpL *mutant compared to Δ*hrpV*, hinting at an additional layer of negative feedback in play, independent of HrpV. Surprisingly, when *hrpL* transcription was reconstituted heterologously in *E. coli*, using plasmids carrying *hrpRS* and the GFP reporter fusion, HrpL was able to repress the activity of its own promoter by approximately 90%. The fact that this occurred independently of the wider DC3000-specific regulatory network led us to conclude that HrpL is able to act sufficiently in negative autogenous control of P*hrpL*.

Making complementary mutations in the *hrp*-box sequence and HrpL DNA-binding domain, we subsequently demonstrated that negative autogenous control by HrpL is dependent on its canonical sigma factor activity at the adjacent *hrpJ* promoter: only when both mutations were acting simultaneously was repression of P*hrpL* completely relieved. Given that the classical ECF sigma factor in *E. coli*, σ^E^, permits the RNAP to occupy DNA upstream of the promoter element, we hypothesised that the P*hrpJ*-bound RNAP-HrpL holoenzyme, likewise, might overlap the distal region of the P*hrpL* UAS and interfere with HrpRS function. In fact, our ExoIII footprint data suggested that RNAP-HrpL occupies a longer 130 nt stretch of promoter DNA, occluding completely the P*hrpL* UAS and partially the site at which the Integration Host Factor (IHF) binds to induce DNA looping. Closer examination of the distal-most region occupied by RNAP-HrpL revealed a near-consensus UP element, an AT-rich sequence with which the C-terminal domain (CTD) of the RNAP α subunit, separated by a flexible linker, can associate in *E. coli*. We have therefore proposed that the α-CTD is the mediator of negative autogenous control by the RNAP-HrpL holoenzyme, although this has yet to be tested experimentally.

As strict EBP-dependent activation usually negates the need for additional regulatory control, direct repression of σ^54^-dependent transcription by other means is very rare. Negative autogenous control by HrpL is therefore not only remarkable in terms of σ^54^-dependent systems, but, given that negative feedback is also introduced by the repressive function of HrpV on HrpS activity, further highlights the apparent importance of fine-tuning T3SS expression in DC3000. Previous studies of negative autogenous control, believed to affect 40% of transcription factors in *E. coli*, showed that it can provide synthetic gene circuits with robustness and rapid response times. However, with this comes the potential fitness cost of reduced transcription factor expression. We therefore explored a range of hypotheses that might explain how decreased expression of the T3SS regulon conveys a positive effect on fitness. Feedback loops can influence the population-level behaviours of pathogens during infection and indeed heterogeneity of T3SS gene expression has recently been shown in *P. syringae*. However, our flow cytometry analyses did not suggest that negative autogenous control impacts significantly on the T3SS expression variance across the population. Similarly, despite previous reports hinting at functional antagonism between the T3SS and the flagellum, another key determinant of plant colonisation, our data did not evidence HrpL-dependent repression of motility.

We instead propose a fitness model whereby an overall limitation of T3SS expression is an advantageous strategy for host-specific pathogens, such as *P. syringae*, that colonise both susceptible hosts and resistant non-host species. A caveat of host-specificity, defined by highly-evolved effector repertoires, is susceptibility to the adaptive immune responses of non-hosts. Given the observations that (i) many *P. syringae* pathovars can colonise non-hosts asymptomatically and (ii) T3SS expression in DC3000 is induced non-specifically by a range of plant exudates, a T3SS-associated fitness trade-off between virulence and immune elicitation is entirely plausible. Thus, when considering the meta-population of DC3000 cells dispersed across a variety of plant hosts, genotypes that impose negative feedback on HrpL expression may experience a positive fitness benefit compared to otherwise more virulent strains. In support of this model, although our genetic studies of negative autogenous control in culture medium do not directly speak to its relevance *in planta*, preliminary data suggest that negative feedback still acts upon P*hrpL* during infection of the *Arabidopsis thaliana* host (our unpublished work).

Using targeted multiple reaction monitoring mass spectrometry (MRM-MS) to determine the relative intracellular and extracellular abundances of four key T3SS-associated proteins (the pilus subunit HrpA1, the harpins HrpZ1 and HopP1, and the effector AvrPto1), we studied the effect of modest increases in HrpL concentration on T3SS activity. Remarkably, only a 2-fold increase in HrpL was sufficient to compromise normal function, resulting in hyper-secretion of the pilus protein HrpA1 and intracellular accumulation, by more than 5-fold, of the three later substrates. We hypothesised that the accumulation of T3SS substrates inside the cell signifies either (i) rate saturation of the translocation step, in which proteins are thought to pass through the pilus channel in an unfolded state, or (ii) an inability to correctly regulate the switch from pilus translocation to substrate translocation, perhaps due to an imbalance in the stoichiometry of T3SS proteins. In the first scenario, the accumulation of surplus, non-secreted substrates might impose a metabolic cost to the cell, an idea supported by the fact that ΔT3SS mutants have a growth advantage over wild-type cells in other bacteria. In the second, the fact that HrpA1 is thought to be an elicitor of plant immunity implies that its release from the cell, whether due to mechanical shearing of excess pilus or complete translocation, would be deleterious in the contexts of both host and non-host plants.

The discovery of negative autogenous control by HrpL advances our knowledge of the regulatory system controlling T3SS gene expression in DC3000 and highlights the importance of exploring the complexity that underlies otherwise well described genetic networks. New insights into the regulation of the T3SS and other bacterial virulence factors promise to inform next-generation interventions for plant disease management. In recent years there has been growing interest in anti-virulence strategies as more evolutionary stable alternatives to crop resistance breeding, whereby the suppression of virulence it supposed to impose a weaker selection pressure for bacterial resistance than inhibiting host colonisation altogether. Promising reports suggest that the T3SS can be readily inhibited by a range of specific anti-virulence chemicals.

